# American Medical Association

**Published:** 1853-06

**Authors:** 


### THE WESTERN JOURNAL.

THIRD SERIES.—VOL. XI.—NO. VI.

LOUISVILLE, JUNE, 1853.

### AMERICAN MEDICAL ASSOCIATION.

(*Concluded from page* 464.)

We copy from a New York paper the following account of the banquet:

### THE MEDICAL DINNER AT METROPOLITAN HALL.

*Grand Entertainment of the Sons of Esculapius in New York.*

The delegates to the convention of the American Medical Association having terminated their anxious labors for the dignity and welfare of the profession, in their sixth annual meeting, held in this city yesterday afternoon, availed themselves of the fraternal and hospitable invitation of the medical faculty of New York, to enjoy a grand entertainment at Metropolitan Hall.

Our daily extended reports of the regular sessional proceedings of the convention, have already conveyed a pretty accurate idea of the venerable aspect, professional worth, and individual respectability, represented from every section of the American continent, in the Bleecker Street Presbyterian Church.

Yesterday's entertainment, however, was a finale worthy of the men who gave, and in every way worthy of the men who partook of it.

Since the arrival of the delegates in this city, the committee of Arrangement and Reception has been under the Presidency of Dr. F. Campbell Stewart, of New York, assisted by the following gentlemen, viz:

Drs. John G. Adams, James R. Wood, John Watson, W. Detmold, Jackson Bolton, Benj. Ogden, Wm. H. Jackson, Edward L. Beadle, Henry D. Bulkley, John H. Griscom, Charles L. Gilman, Wm. H. Van Buren, George F. Woodward, Wm. Rockwell, Thomas Ward, Charles D. Smith, B. Fordyce Barker, Robert Watts, S. Conant Foster, J. W. G. Clements, Isaac E. Taylor, John O. Stone, George A. Peters, Jas. S. Cooper, Charles Herschel, Gurdon Buck, and Jared Linsley.

How they discharged their duties towards the press and public is already known. The medical faculty of this city, having determined upon receiving their brethren, entrusted the entire matter to their hands. An entertainment in Metropolitan Hall was determined upon, at which the practitioners of the city and in the forest, the mountain and the prairie, the North and South, the East and West, and of all the districts and territories of our mighty confederation, could sit down and enjoy the hospitality of their brethren residing in the great Empire City of the whole.

The dinner took place at seven o'clock yesterday afternoon, in Metropolitan Hall. The guests were shown into the ample reception room immediately upon their arrival, and there a sectional committee attended to take charge of hats, cloaks, &c., which were duly deposited in numbered stalls.

From this they were shown to the main hall of the building, where the dinner was served up.

Upon entering we found the platform—familiarized to our eyes and ears by the personal bearing and artistic performances of Jenny Lind, Sontag, Alboni, and Julien—decorated by the most costly flowers and evergreens, of which a vast pyramid, capped with roses, occupied the centre. This floral arrangement, which had been entrusted to Mr. Thomas Dunlap, was most refreshing.

Immediately behind were seats for Dodsworth's Band, which attended in full force, and performed several magnificent airs during the evening.

From the platform to the door of entrance, the hall, the great space, was divided by fourteen tables, extending in straight lines, which were covered with the most rare and exquisite delicacies. Immediately in front of the platform two long tables swept around, at which the members of the press were accommodated. Opposite to each guest there was placed a bill of fare, printed upon rich white satin, trimmed with a heavy border of azure blue. It is unnecessary to say that the tables "groaned with the weight of the feast," when we mention that soups, oysters, cold dishes, game, confectionary, ices, fruits, tea, coffee, &c., were served up in New York abundance.

The galleries were filled with ladies, who were admitted by ticket from the committee of Arrangements. When the dinner was served up, and the host of waiters and diminutive pages, dressed with turbans, in Eastern costume, were at their posts, the effect was almost overpowering. No assemblage in America, perhaps in the world, ever met combining so much moral worth, self-denial in the cause of humanity, disinterested exertion for the advancement of science, and a more thorough identity of educated alliance to our free institutions than this did.

The committee of Arrangements entered the hall, preceded by Dr. Stewart, and followed by the guests. Upon their entrance the band played

"A march from the opera of the Black Domino."

About seven hundred members followed, who were received by the courteous and inspiriting recognition of nearly five hundred ladies in the galleries.

Doctor Jonathan Knight, of Connecticut, President of the American Medical Association, took the chair. Immediately upon his right and left we observed the Hon. Judge Oakley, the Hon. the Recorder Tillou, Ex-Mayor Kingsland, Rev. Dr. Peet, Dr. J. W. Francis, Rev. Dr. Osgood, Peter Cooper, Esq., and many of our citizens most distinguished at the bar, upon the judgment seat, in the pulpit, and in the counting room.

The dinner was served up by Mr. J. J. Moffatt, of No. 579 Broadway, assisted by Mr. Pentin, having under their charge an army of waiters. The cost of this entertainment amountnd to from $500 to $25 each upon our New York doctors, a fact which we mention in order that our readers may have an idea of its grandeur. Towards the conclusion of the entertainment, a brace of doves flew from a monster pie placed upon one of the tables, and having hovered around the brilliant hall for a long time in search of a resting place, deposited the emblem of peace and harmony over the heads of a group of ladies in the hall, who were conducted to seats in the side aisle of the main hall, by Dr. Hayes, of Pennsylvania, amidst loud cheers.

After the carving of turkeys, ducks, wild fowl, lamb, roast beef, &c., had proceeded with an accuracy which must have been grateful to the manes of John Hunter, and when the doctors had arrived at that *spiritu-elle* elevation from which they could look down with professional contempt upon the "oxygenated bitters," and "nervous antidotes" of this morning, the intellectual and patriotic entertainments of the evening commenced.

The President then announced the first regular toast, as follows:

"The President of the United States"—(Air, Hail Columbia.)

When the cheering with which this was received, had subsided, the second was read:

"The Governor of the State of New York."

The third regular toast was:

"The American Medical Association—it has passed through the diseases of infancy with constitution unimpaired—a manhood of strength and usefulness awaits it."

This toast was responded to by Dr. Knight, who spoke substantially as follows:

It accords with my feelings to respond to the sentiment which you have proposed, although I wish the duty had been imposed upon those who are more capable of performing it. The medical faculty of this city have, since our arrival, treated us with the most liberal hospitality, and extended to us the open hand of brotherhood. Your hospitality has extended to us its generous care by night and by day; our paths have been strewed with the tokens of your kind consideration. Indeed, so full has been the enjoyment of the members, so powerfully has it been expressed, I had serious apprehensions lest this medical association should resolve itself into a permanent body and hold perpetual session in the city of New York. (Laughter and applause.) And I am sure now, after witnessing the sight presented to our view to-night, (looking at the ladies in the gallery,) that, whatever doubts I might have entertained, those apprehensions would ripen into certainty. I know not what the feelings of your good citizens might have been at this sudden visit of three or four hundred physicians, in addition to the number you have already here. I recollect very well that upon the taking of the census of this city some years ago, the number of inhabitants was stated at 100,000, with possibly a proportion of 150 medical men; but with the increase of population to 500,000, or 600,000, there have been added some five or six hundred gentlemen of the profession. Notwithstanding what has been said of the medical profession of the present day, in publications, in private conversation, in reports to this or that learned body, I believe it has steadily advanced. It has been said that the medical profession has degenerated, but I believe it has progressed with all other sciences, and I have no hesitation in saying that the time will come when the labors of the physicians of the present day will be appreciated as their merits deserve. But it was not my intention to enlarge upon this, or any other subject. I arose merely, in the name of the Medical Association, to return you their kind acknowledgements for the liberal hospitality you have treated them with.

After the conclusion of Dr. Knight's remarks, the fourth regular toast was proposed:—

"Divinity, Law, Medicine—Three graces, all of which combined, support each other."

To this toast, Rev. Mr. Osgood responded, in a few brief and appropriate remarks.

He said that he learned when a boy to do as the doctor said, and he never forgot the old habit. But for the first time in his life, the doctor had got him into trouble, instead of helping him out of it. He spoke of the intimate relation between the professors of medicine and divinity, and illustrated his points by some amusing anecdotes. He ended by exhibiting the two professions as interpreters of the same heavenly mercy, and gave the following sentiment, after speaking of medicine as nature evangelized:—

"Medicine and Divinity—The two stood together in the beginning, when science was darkened by superstition; they shall stand nearer together at the end, when science and faith shall be recognized as different, but harmonious, aspects of the same divine wisdom and goodness."

In compliance with the general request, Judge Oakley arose, but merely returned his thanks for the honor which had been conferred on the profession of which he was a member.

Dr. Francis was next called out, and was received with repeated applause. He spoke in substance as follows:

Gentlemen, and Members of the Medical Association:—I don't know exactly upon what topic I shall address you. I believe the sentiment involves law, divinity and medicine. I am taken somewhat by surprise, and particularly when I look around this hall, and see this vast medical faculty. I wish that an individual of more potentiality had been called upon to respond to this toast; but as the matter is now before us, I may say that while I listened to the address of the Rev. Mr. Osgood, D. D., and of Judge Oakley, L. L. D., it appears that nothing but the subject of medicine is left for me. But I shall take up the whole three subjects—lawyers, divines, and doctors. I shall say very little on each, however. It is evident, Mr. President, that from the first organization of society; from the foundation of the first hamlet or village, down to the establishment of a mighty State like this, order must have obtained, or such a condition of things could not have existed. It must therefore be certain, that law existed, or order, or government, at a very early period. And I take it that the history of society shows that order, government, discipline, regulation, and preservation of law, is indispensable. Therefore, law exists with man from the beginning, and continues as he advances to that perfect state to which he is capable of arriving. Divinity is an inherent principle in man, for I contend that man, in his early state, is religious; and in his primordeal condition, was endowed with religious principles; and hence, I affirm, the religious principle is innate in man. Now that we have seen law well dispensed, we find religion engrafted on law, and that both these branches of profound science have always gone hand in hand. The history of eminent men abroad shows you the connection between law and divinity. But, gentlemen, there is another point to which I would call your attention. With the principles of law and religion, we find, in the earliest state of society, men practicing medicine; the priest and prescriber went hand in hand together; and hence, law, physic and divinity, are one harmonious trinity.

After a few further remarks, Dr. Francis concluded by paying a compliment to Dr. Wellford, late President of the Association, and proposed, as a toast, his health and prosperity.

After his health was drank, Dr. Wellford returned his thanks, and responded to the toast in a brief and appropriate speech:

"Union—Upon it depends the safety of individuals, the strength of States, the existence of nations. The physicians of America, will always be found among its warmest supporters."

The next toast was as follows:

"The Union of Science and Literature.—A happy marriage, the fruits of which are no where seen to a better advantage than in our American Holmes."

To this, Dr. Wendell Holmes responded, by reading a humerous and witty poem which he had prepared for the occasion, and which excited considerable merriment among the company.

The next toast was—

"Woman—Who so well as the physician can appreciate her varied excellence!"

